# Intersectorality, key to address Social Health Inequalities

**DOI:** 10.1590/1518-8345.0000-3124

**Published:** 2019-04-29

**Affiliations:** 1 Universidad Autónoma de Madrid, Faculdad de Medicina, Departamento de Enfermería, Madrid, Espanha. Instituto Interuniversitario “Investigación Avanzada sobre Evaluación de la Ciencia y la Universidad” (INAECU), Madrid, Espanha. Instituto de Investigación Sanitaria Puerta de Hierro Majadahonda (IDIPHIM), Madrid, Espanha; Instituto Interuniversitario Investigación Avanzada sobre Evaluación de la Ciencia y la Universidad, Madrid, Espanha; Instituto de Investigación Sanitaria Puerta de Hierro Majadahonda, Madrid, Espanha


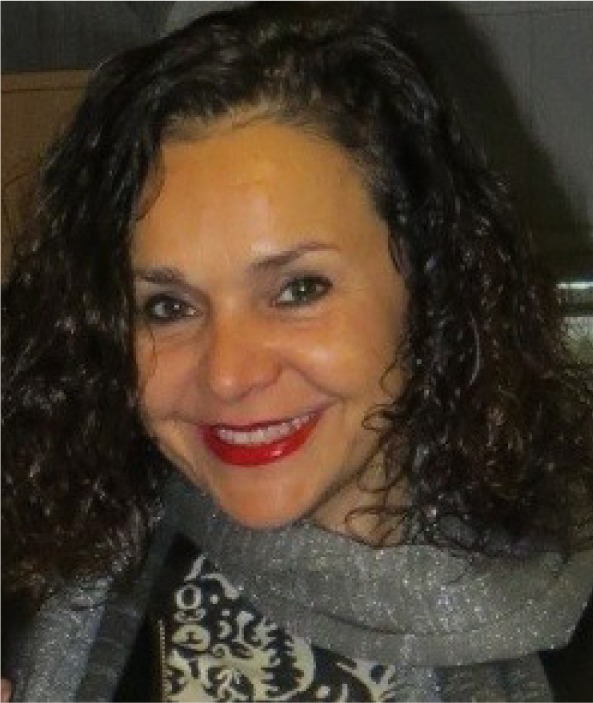


The conceptual framework for action on the social determinants of health^(1)^, showed up the truss that we must necessarily incorporate to understand how social health inequalities (SHI) are produced. From this analysis are given the keys to intervention, being the intersectoriality one of the most crucial and strategic, and that inexorably implies that the health sector can never think that it is the protagonist to address the SHI, but that a distributed leadership is essential among the different sectors (health, social, education, environment, urban planning). However, it is also essential to understand intersectoriality as a technical, administrative and political process that involves negotiation and distribution of power, resources and capacities (technical and institutional) between the different sectors; and that, therefore, not only demand of a social vision or a political intention of governance, but also of the development of new management capacities and new institutional commitments^(2)^.

A recent scoping review^(3)^ revealed that, in the intersectoral actions, and because of the resistance generated by a loss of organizational autonomy, a sectoral logic prevails that hinders cooperation, the distribution of responsibilities and an operational action. In addition, governments do not promote the decentralization of power and the empowerment of civil society. Likewise, the policies are fragmented and with discontinuity in their implementation, management and inspection. Finally, gender and ethnicity, as axes of inequality, as well as poverty, remain “accounts pending” to implement intersectoral projects. Nevertheless, it also showed that successful intersectoral actions are presented in combination with strong community participation strategies.

This leads us to think that Health Promotion (increases the control of the determinants of health by citizens), the creation of networks (horizontality, interaction, exchange, mutual respect, feeling of belonging and integration and management sharing of knowledge contribute to the development of capacities), and Health Assets Model (resources that improve the capacity of the communities to maintain and promote their health which constitute the Social Capital) are the keys to face the SHI from an integration intersectoral approach that drives “Health in all policies”, allowing the definition of policies and programs together among all sectors, and whose starting point is cross-cutting management.

The National Strategy of Equity in Health of the Ministry of Health, Social Services and Equality (2012) of the government of Spain, marked four lines of work, the second being "promoting and developing intersectoral knowledge and tools", through the creation of sectoral bodies, the inclusion of intersectoral objectives in all health plans, equity training in the health sector and awareness of the importance of SHI.

The intersectoriality cannot be contemplated without Public Engagement, which entails a new model of governance. In this regard, the Madrid City Council launched in 2017 the Plan "Madrid City of Care"(4), a strategy of intersectoral action that puts the sustainability of life at the heart of municipal action, seeking a new relationship with citizens, from the ethics of care, focused on both community empowerment and respect for the autonomy and diversity of people. It is still early to have results from this ambitious plan, but very encouraging experiences have already been set in motion (design of school environments, prevention of unwanted loneliness, healthcare inclusion of all citizens, community intervention in unemployed men, etc.) that will surely make Madrid a city with greater equity and consequently with more health.

To conclude, I would like to emphasize that an intersectoral approach cannot be carried out without the social participation of all stakeholders. Intersectoriality and social participation are an indissoluble binomial to resolve SHI.
